# War and Disease: Biomedical Research on Malaria in the Twentieth Century

**DOI:** 10.3201/eid1507.090451

**Published:** 2009-07

**Authors:** Eugenia Tognotti

**Affiliations:** University of Sassari, Sassari, Italy

**Keywords:** malaria, history, biomedical research, World War II, book review

War and Disease is a fascinating historical account of the discovery of drugs effective against malaria, one of the great scourges of humankind. The author, Leo B. Slater, makes good use of his expertise as a historian of biomedical science and technology. He provides a meticulous reconstruction of the manner in which the scientific community, in the midst of World War II, established an antimalarial program, which was to biomedical research what the Manhattan Project was to the physical sciences. At a time when industrialized nations are involved in the effort to find solutions to the ongoing global health catastrophe that malaria is today, this volume is a timely and valuable contribution.

Malaria’s effects have long been at the center of colonial expansion and war. The disease became a focus of research in the late nineteenth century. France and Great Britain had expanded their colonies into areas of the world where malaria was the most severe and debilitating of the parasitic tropical diseases—a factor that limited the colonial governments’ exploitation of natural resources. Malariologic research thrived in these countries and in Italy, where the disease was not a colonial problem but an endemic scourge and overwhelming obstacle to development, just as it is in many developing countries today. From 1880 through 1898 research carried out by such prominent scientists as Charles-Louis-Alphonse Laveran, Ronald Ross, Angelo Celli, Ettore Marchiafava, and Giovanni Battista Grassi led to the discovery of the malaria transmission cycle**.** The neurologist, Camillo Golgi, who shared the 1906 Nobel Prize for his work on the structure of the nervous system, studied the reproductive cycle of the parasite (*Plasmodium* spp*.*) and elucidated the synchronicity between the symptoms of recurrent chills and fever and the rupture and release of merozoites into the blood. These findings offered an explanation for the effectiveness of treatment with quinine, which had been used empirically as a generic febrifuge since the 17th century. The alkaloid remained the only effective option for treating malaria until the 1930s.

During World War II the disease reached epidemic proportions among American troops fighting the Japanese in the South Pacific. Quinine was the main line of defense, but the supply of quinine was cut off by Japanese military conquest. The development of new antimalarial drugs became a major subject of research. Three chapters of the book—Preparing for War, Preparation and Coordination, Trust and Transition—focus on the comprehensive research program, which integrated exceptional technical and scientific expertise into a massive organizational and cooperative effort. Funded by the United States Office of Scientific Research and Development, this program screened ≈14,000 compounds for antimalarial efficacy. Clinically approved Atabrine (quinacrine) became the drug of choice in 1943, and, shortly after the war, chloroquine was identified, which has had an enduring influence on antimalarial chemotherapy. The wartime effort, Slater argues, was essential to the development of the US National Institutes of Health. Making a critical point, however, he suggests that although the wartime antimalarial program is an excellent model for future large-scale biomedical research projects, it is “a potential example of how not to pursue public health research for impoverished civilians” (p. 2). The transfer of research results into health benefits for the most vulnerable persons in malarial lands was not on the agenda. The widespread usefulness of a drug such as chloroquine was only a side effect.

